# Gene-environment interaction effects on lung function- a genome-wide association study within the Framingham heart study

**DOI:** 10.1186/1476-069X-12-101

**Published:** 2013-12-01

**Authors:** Shu-Yi Liao, Xihong Lin, David C Christiani

**Affiliations:** 1 Harvard School of Public Health, 665 Huntington Avenue, Boston, MA 02115, USA; 2 Harvard Medical School, 665 Huntington Ave, Boston, MA 02115, USA

## Abstract

**Background:**

Previous studies in occupational exposure and lung function have focused only on the main effect of occupational exposure or genetics on lung function. Some disease-susceptible genes may be missed due to their low marginal effects, despite potential involvement in the disease process through interactions with the environment. Through comprehensive genome-wide gene-environment interaction studies, we can uncover these susceptibility genes. Our objective in this study was to explore gene by occupational exposure interaction effects on lung function using both the individual SNPs approach and the genetic network approach.

**Methods:**

The study population comprised the Offspring Cohort and the Third Generation from the Framingham Heart Study. We used forced expiratory volume in one second (FEV_1_) and ratio of FEV_1_ to forced vital capacity (FVC) as outcomes. Occupational exposures were classified using a population-specific job exposure matrix. We performed genome-wide gene-environment interaction analysis, using the Affymetrix 550 K mapping array for genotyping. A linear regression-based generalized estimating equation was applied to account for within-family relatedness. Network analysis was conducted using results from single-nucleotide polymorphism (SNP)-level analyses and from gene expression study results.

**Results:**

There were 4,785 participants in total. SNP-level analysis and network analysis identified SNP rs9931086 (P_interaction_ =1.16 × 10^-7^) in gene *SLC38A8*, which may significantly modify the effects of occupational exposure on FEV_1_. Genes identified from the network analysis included *CTLA-4, HDAC*, and *PPAR-alpha*.

**Conclusions:**

Our study implies that SNP rs9931086 in *SLC38A8* and genes *CTLA-4*, *HDAC,* and *PPAR-alpha*, which are related to inflammatory processes, may modify the effect of occupational exposure on lung function.

## Background

Chronic obstructive pulmonary disease (COPD) is defined by decreased lung function, commonly measured by forced expiratory volume in one second (FEV_1_) and the ratio of FEV_1_ to forced vital capacity (FVC). Cigarette smoking is the most important environmental risk factor for COPD. However, COPD development in smoking populations is not universal. In a study conducted by the American Thoracic Society [1], approximately 15% of COPD may be attributable to workplace exposures; a similar estimate was reported in a more recent study [2]. Indeed, an association between COPD or poor lung function and occupational exposure to dust, gas, and fumes has been reported in both industry-based studies [3-6] and community-based [7-11] cross-sectional studies. In addition, mounting evidence indicates that genetic factors contribute to COPD. In fact, the disease aggregates in families [12], and genome-wide association studies (GWAS) [13-17] have identified single nucleotide polymorphisms (SNPs) that are associated with COPD or lung function.

To date, few study has comprehensively examined combined genetic and environmental effects on lung function. GWAS on lung function have considered genetic effects alone; a recent review [18] showed that most GWAS findings do not replicate consistently, which may imply a role played by environmental exposures and gene-environmental interactions. Studies of occupational exposure have only investigated the main effect of exposure on lung function. Therefore, some disease susceptibility genes may be missed due to low marginal effects, despite being involved in the disease process through interactions with environmental exposures [19]. Further, the majority of gene-environment interaction studies of lung function or lung function has focused on gene by smoking interaction [20-23]. However, no reports describe gene by occupational exposure interactions. In addition, most researchers investigated SNPs or genes through either *in silico* statistical methods or by gene expression experiments, but did not integrate these results. Thus, an integrated, genome-wide gene-environment interaction study can more readily identify disease susceptibility genes when environmental factors may also be contributing.

The purpose of this study was to investigate gene by occupational exposure interactions on FEV_1_ and FEV_1_/FVC thorough SNP-level analysis and network analysis. Specifically, we first performed a GWAS using data from the Framingham Heart Study (FHS) to identify SNPs that interact with occupational exposure using a population-specific job exposure matrix (JEM) to affect (FEV_1_) and ratio of FEV_1_ to FVC. We next combined our GWAS SNP results with gene expression results to build a network of biological processes that are driven by networks and not by individual genes. The findings from these analyses uncovered several SNPs/Genes that may offer avenues for future functional studies of genes contributing to lung function changes following occupational exposures.

## Methods

### Study population

Our study population derives from the FHS [24], which includes only Caucasians. This study has recruited participants since 1948; there have been three generations of participants: the Original Cohort, their Offspring, and the Third Generation. Spirometry measurements, detailed medical history, physical examinations, and laboratory tests were done approximately every two years. We used the 4,785 participants with complete spirometry phenotypes, occupational information, genotypes, and related covariates from the Offspring Cohort and the Third Generation Cohort.

### Ethics statement

Written informed consents were provided by all participants. Protocols were approved by local institutional review boards.

### Spirometry phenotypes and covariates

Spirometry from participant lung function Exam 8 and the Third Generation Exam were used in our study. We used the FEV_1_ and FEV_1_ ratio (FEV_1_/FVC) as continuous outcomes. Age, gender, height (inch), pack-years, and smoking status were used as covariates in our analysis. Smoking status (never, former, and current smokers) was coded as dummy variable.

### Genotyping and quality control

Genotyping for 500,568 SNPs was conducted with approximately 550 K SNPs using the Affymetrix 500 K mapping array plus Affymetrix 50 K supplemental array in 9,237 subjects from the three generations of participants. We used 4,785 subjects from two generations in our study. A quality test was conducted using the PLINK software (version1.06, http://pngu.mgh.harvard.edu/~purcell/plink/). A total of 499 individuals with genotyping call-rate <95% were deleted, and the genotyping rate in the remaining individuals was 98.6%. We conducted the Hardy-Weinberg test for all SNPs, and found 19,546 SNPs had a p-value <1 × 10^-6^. These SNPs showed an obvious deviation from the 45-degree line of a QQ plot and were excluded from our analysis. A total of 34,110 SNPs had a per-SNP missing rate >5% among all subjects and were excluded. We also excluded 146,203 SNPs with minor allele frequency lower than 5% in our study subjects. After filtering, 300,709 SNPs remained for analysis.

### Occupational exposure

We used a job exposure matrix (JEM) for occupational exposure assessment. Occupational exposure was classified as high versus low likelihood for dust exposure (coded as 1 and 0) according to their job categories based on a questionnaire in Offspring Exam 8 and the Third Generation Exam (Table 1). The questionnaire asked, “Using the occupation coding sheet choose the code that best describes your occupation”. There are 29 job categories (exclude retired) on the FHS occupation coding sheet, and we classified 4 of them as highly likely dust exposure including factory/assembly/mechanic, skilled labor, general labor, and heavy labor, as modified from UCSF COPD JEM (January, 2009 revision) [25].

**Table 1:** Job categories for dust exposure classification

**Dust exposure group**	**Job category**
Highly likely dust exposure	Factory/assembly/mechanic
	Skilled labor (e.g., plumber, carpenter, painter, hairdresser)
	General labor (e.g., custodian, delivery, mailman, truck driver)
	Heavy labor (e.g., construction, landscaping)
Less likely dust exposure	Nurse/medical personnel
	Laboratory technician
	Physical/occupational/speech therapist
	Police/fire/security/military
	Restaurant/food worker
	Homemaker
	Self-employed business owner
	M.D./dentist/scientist/research
	Lawyer/judge
	Psychologist/social worker/mental health counselor
	Engineer/computer science
	Banker/accountant
	Manager/consultant (e.g., production manager)
	Administrative (e.g., personnel)
	Educator
	Secretary/clerk/data entry
	retail/cashier
	Sales/marketing/insurance
	Realtor
	Writer/editor/artist/graphic designer/craftsperson
	Musician
	Clergy (minister, priest, rabbi)
	Sports pro/coach/exercise instructor/other
	Statistician
	Student

### Statistical analysis

#### SNP-level analysis

The family-based cohort was analyzed using linear regression-based generalized estimating equations implemented in the GWAF package for R [26] to account for within-family relatedness. The within-pedigree correlation matrix was modeled using an exchangeable working correlation matrix. We analyzed each SNP separately using FEV_1_, and FEV_1_/FVC as outcomes. For each SNP, we included age, gender, height (inch), pack-years, smoking status, occupational exposure status, and tested for the main SNP effect. We next fit an interaction model by adding an SNP by occupational exposure status interaction to the model, and tested for the SNP-exposure interaction.

#### Network analysis

To identify important networks among the genes that interact with occupational exposure, we used Metacore software (GeneGo, St Joseph, MI, USA) tools to build networks using the gene lists. One gene list was obtained from a previous study [27], in which human airway epithelial cells obtained from 6 normal individuals were exposed to coarse, fine, or ultrafine particulate matter for 6 and 24 hours before gene expression was assessed. The authors reported 71 unique genes altered by the particulate matter (PM). To the best of our knowledge, no other studies have focused on the effects of dust exposure or PM on gene expression in human airway cells. Another list of genes was generated from our results. We chose SNPs whose interactions with occupational exposure had p-values smaller than 10^-3^ (around top 0.1% of SNPs), and mapped these SNPs to genes to generate a gene list. Gene annotation was performed using the gene prediction track “RefSeqGenes” in the UCSC browser (http://genome.ucsc.edu).

## Results

This study comprised 4,785 participants, with 1,247 participants from the Offspring Cohort and 3,538 participants from the Third Generation. Among them, 4,238 participants were classified in the less likely dust exposure group and 547 participants in the highly likely dust exposure group.

Table 2 summarizes the characteristics for participants by both exposure group and cohort. Mean FEV_1_ was higher in the group with highly likely dust exposure, but the mean FEV_1_/FVC was similar in the two groups overall. However, the proportion of COPD cases (defined by FEV_1_/FVC less than 70%) was higher in the group with highly likely dust exposure. Lung function was higher and the proportion of COPD cases was lower in the Third Generation as compared to the Offspring Generation, most likely attributable to the younger age of participants in the Third Generation.

**Table 2:** Characteristic of participants, stratified by cohort and dust exposure groups

	**Offspring**		**Third generation**		**Total**	
	**(n = 1247)**		**(n = 3538)**		**(n = 4785)**	
	**Less likely dust exposure**	**Highly likely dust exposure**	**Less likely dust exposure**	**Highly likely dust exposure**	**Less likely dust exposure**	**Highly likely dust exposure**
	**(n = 1126)**	**(n = 121)**	**(n = 3112)**	**(n = 426)**	**(n = 4238)**	**(n = 547)**
Male, n (%)	444 (39.43)	100 (82.64)	1298 (41.71)	378 (88.73)	1742 (41.10)	478 (87.39)
FEV_1_, L ± SD	2.71 ± 0.78	3.09 ± 0.75	3.54 ± 0.78	4.02 ± 0.71	3.32 ± 0.86	3.81 ± 0.82
FEV_1_/FVC, % ± SD	0.73 ± 0.07	0.72 ± 0.08	0.78 ± 0.06	0.77 ± 0.06	0.77 ± 0.07	0.76 ± 0.07
FEV_1_/FVC < 70%, n (%)	337 (29.93)	43 (35.54)	318 (10.22)	49 (11.50)	655 (15.46)	92 (16.82)
Age, years ± SD	62.83 ± 8.02	60.48 ± 7.87	39.81 ± 8.58	41.03 ± 8.57	45.93 ± 13.21	45.33 ± 11.66
Height, inches ± SD	65.81 ± 3.76	67.99 ± 3.46	66.90 ± 3.65	69.29 ± 2.98	66.61 ± 3.71	69.01 ± 3.14
Pack-years^*^ ± SD	21.40 ± 21.58	30.73 ± 23.32	12.56 ± 13.61	19.12 ± 15.90	15.50 ± 17.19	22.07 ± 18.74
Smoking status, n (%)						
Never smokers	501 (44.49)	36 (29.75)	1854 (59.58)	177 (41.55)	2355 (55.57)	213 (38.94)
Former smokers	522 (46.36)	58 (47.93)	789 (25.35)	115 (27.00)	1311 (30.93)	173 (31.63)
Current smokers	103 (9.15)	27 (22.31)	469 (15.07)	134 (31.46)	572 (13.50)	161 (29.43)

*Pack-years mean and standard deviation calculated among current and former smokers.

### SNP-level analysis

The genome-wide analysis for 300,709 SNPs (Bonferroni correctionα = 1.66 × 10^-7^) detected a significant interaction effect of SNP rs9931086 in gene *SLC38A8* on chromosome 16q23.3 and occupational exposure on FEV_1_ (P_interaction_ = 1.16 × 10^-7^). Further, gene-environment interaction effects of SNPs rs3889785 and rs4234966 in the *MARCH1* gene and SNPs rs17508671, rs17430621, and rs17508706 in the *ZNF804A* gene and occupational exposure on FEV_1_ also had small P values (all P_interaction_ < 3.04 × 10^-5^). The genomic inflation factor value of 1.045 calculated from the QQ plot suggested the p-values were appropriately distributed. The results for the top 30 SNPs with strongest occupational exposure interaction effects on FEV_1_ are shown in Table 3 and the Manhattan plot is shown in Figure S1 (see Additional file 1).

**Table 3:** **Top 30 strongest SNPs interacting with occupational exposure to affect FEV**_**1**_

**SNP**	**Chr**	**Position**	**Band**	**Gene**	**All1**	**All2**	**MAF**	**β**_**SNP**_	**P**_**SNP**_	**β**_**interaction**_	**P**_**interaction**_
rs9931086	16	84046105	q23.3	SLC38A8	A	C	0.19	27.70	2.53E-02	-214	1.16E-07*
rs11252277	10	4065704	p15.1		G	A	0.09	2.90	1.05E-01	-263	6.32E-07
rs3889785	4	165097529	q32.3	MARCH1	A	C	0.13	-2.02	1.69E-01	237	1.05E-06
rs7098287	10	4050329	p15.1		G	C	0.09	3.60	4.88E-02	-247	2.47E-06
rs32977	5	14119057	p15.2		A	G	0.06	5.50	7.38E-03	-310	3.98E-06
rs17508671	2	185491847	q32.1	ZNF804A	A	G	0.16	-0.27	8.32E-01	-173	4.45E-06
rs2544785	19	49085710	q13.33	SULT2B1	C	T	0.16	0.03	0.983642	172	4.57E-06
rs4234966	4	165078580	q32.3	MARCH1	C	G	0.12	-1.92	1.88E-01	217	6.35E-06
rs32978	5	14119645	p15.2		C	T	0.05	5.05	1.75E-02	-302	6.63E-06
rs32979	5	14119775	p15.2		T	C	0.05	5.05	1.75E-02	-302	6.63E-06
rs17645582	6	82797317	q14.1		C	T	0.07	-1.07	5.75E-01	208	6.89E-06
rs4932559	15	92133594	q26.1		A	G	0.36	-1.62	0.081898	132	7.10E-06
rs745926	2	120482057	q14.2		T	G	0.41	-1.13	2.24E-01	136	9.90E-06
rs1561577	15	89146968	q26.1		A	G	0.07	-3.17	0.104592	224	1.16E-05
rs6445707	3	54881562	p14.3	CACNA2D3	G	A	0.18	0.40	7.47E-01	166	1.17E-05
rs2544784	19	49085733	q13.33	SULT2B1	A	T	0.16	0.00	0.997879	161	1.25E-05
rs17110400	14	26276467	q12		C	T	0.09	-0.31	0.855973	265	1.53E-05
rs10231843	7	103884705	q22.2		C	G	0.27	-1.10	3.09E-01	160	1.64E-05
rs17430621	2	185492469	q32.1	ZNF804A	A	T	0.16	0.02	9.85E-01	-165	1.89E-05
rs163814	5	14122497	p15.2		C	T	0.05	5.43	1.16E-02	-285	1.94E-05
rs477920	1	74767844	p31.1	FPGT TNNI3K	A	G	0.17	0.23	8.55E-01	-183	2.04E-05
rs6972824	7	157473222	q36.3	PTPRN2	C	A	0.11	2.38	8.22E-02	-196	2.30E-05
rs6124623	20	42746332	q13.12	JPH2	C	G	0.14	1.34	3.00E-01	-172	2.34E-05
rs2832081	21	30152992	q21.3		C	A	0.31	-2.33	0.01353	137	2.36E-05
rs7356986	6	12301462	p24.1		G	A	0.11	-1.68	3.20E-01	202	2.36E-05
rs41328144	1	166319711	q24.1		C	T	0.13	-0.68	6.35E-01	155	2.54E-05
rs2803543	10	88301331	q23.2		G	A	0.10	-2.35	1.49E-01	224	2.90E-05
rs163813	5	14122154	p15.2		G	A	0.05	5.00	1.88E-02	-281	2.96E-05
rs17508706	2	185524591	q32.1	ZNF804A	G	A	0.16	-0.14	9.12E-01	-162	3.04E-05
rs4130671	7	119277137	q31.31		A	C	0.49	-1.37	1.27E-01	122	3.71E-05

SNP-estimates are based on an additive model. Beta-estimates represent the increase or decrease FEV_1_ in mL.

*Significant after Bonferroni correction for testing 300,709 SNPs (α = 1.66E-07).

Chr: Chromosome; All1: allele1; All2: allele2.

For gene by occupational exposure interaction effects on FEV_1_/FVC, although no SNP reached the Bonferroni corrected significance level (α = 1.66 × 10^-7^), SNPs rs7297210, rs6486961, rs7297431, and rs1875467 in gene *AEBP2* and SNPs rs10751811, rs2173524, and rs11595576 in *ADARB2* had small P values (all P_interaction_ < 4.32 × 10^-5^). In addition, SNP rs7314308 had small P values for both the main genetic effect (P_SNP_ = 5.43 × 10^-5^) and the gene by occupational exposure effect (P_interaction_ = 1.20 × 10^-5^). The genomic inflation factor value of 1.043 suggested the p-values were appropriately distributed. The results for the top 30 SNPs with strongest occupational exposure interaction effects on FEV_1_/FVC are shown in Table 4 and the Manhattan plot is shown in Figure S2 (see Additional file 1: Figure S2).

**Table 4:** **Top 30 strongest SNPs interacting with occupational exposure to affect FEV**_**1**_**/FVC**

**SNP**	**Chr**	**Position**	**Band**	**Gene**	**All1**	**All2**	**MAF**	**β**_**SNP**_	**P**_**SNP**_	**β**_**interaction**_	**P**_**interaction**_
rs17051547	4	53421172	q12		C	A	0.13	-0.44	3.31E-02	2.84	2.86E-06
rs17051550	4	53425943	q12		C	G	0.11	-0.38	8.06E-02	2.91	6.01E-06
rs11671079	19	29900846	q12	LOC284395	G	A	0.46	-0.10	0.397666	1.55	8.65E-06
rs2804576	10	113775409	q25.2		A	G	0.13	0.34	9.56E-02	-2.83	1.01E-05
rs7314308	12	59684714	q14.1		G	A	0.19	0.65	5.43E-05	-2.09	1.20E-05
rs7297210	12	19630207	p12.3	AEBP2	T	C	0.40	0.18	2.22E-01	-1.62	1.53E-05
rs2655364	18	26229339	q12.1		T	A	0.46	-0.20	1.43E-01	1.76	1.55E-05
rs4322259	1	64339246	p31.3	ROR1	C	G	0.35	0.21	1.59E-01	-1.72	1.61E-05
rs1504108	18	26224965	q12.1		A	G	0.45	-0.20	1.56E-01	1.76	1.82E-05
rs2655360	18	26231390	q12.1		T	C	0.45	-0.20	1.54E-01	1.73	2.23E-05
rs987124	18	26217351	q12.1		G	A	0.46	-0.20	1.38E-01	1.71	2.54E-05
rs9878581	3	37969695	p22.2	CTDSPL	C	G	0.13	-0.37	6.85E-02	2.23	2.92E-05
rs10751811	10	1687171	p15.3	ADARB2	A	G	0.36	-0.17	2.46E-01	1.53	3.06E-05
rs6486961	12	19626815	p12.3	AEBP2	A	G	0.40	0.13	3.60E-01	-1.59	3.13E-05
rs6941466	6	168929950	q27	SMOC2	T	C	0.22	-0.21	2.54E-01	2.03	3.21E-05
rs7297431	12	19600232	p12.3	AEBP2	T	C	0.45	0.15	2.75E-01	-1.59	3.30E-05
rs10770501	12	19683476	p12.3		G	A	0.46	0.16	2.51E-01	-1.57	3.35E-05
rs537693	6	159662136	q25.3	FNDC1	G	A	0.12	-0.11	6.18E-01	2.28	3.36E-05
rs2173524	10	1687799	p15.3	ADARB2	T	G	0.35	-0.18	2.46E-01	1.51	3.41E-05
rs1105791	9	81179317	q21.31		C	A	0.19	-0.36	0.026841	1.91	3.83E-05
rs1875467	12	19622345	p12.3	AEBP2	C	A	0.46	0.13	3.43E-01	-1.54	4.06E-05
rs9888671	15	26277939	q12	LOC100128714	C	T	0.13	0.17	3.79E-01	2.01	4.09E-05
rs12141717	1	55388480	p32.3		G	A	0.29	-0.08	5.89E-01	1.80	4.24E-05
rs11595576	10	1688497	p15.3	ADARB2	T	C	0.36	-0.16	2.96E-01	1.53	4.32E-05
rs5996665	22	24469115	q11.23	CABIN1	G	A	0.08	0.35	8.54E-02	-2.65	4.41E-05
rs7078873	10	61787587	q21.2	ANK3	T	A	0.38	0.30	3.34E-02	-1.62	4.49E-05
rs4938871	11	57329402	q12.1	UBE2L6	C	A	0.26	-0.36	3.28E-02	1.90	4.63E-05
rs1885166	9	17610211	p22.2	SH3GL2	T	C	0.39	0.10	0.487458	-1.70	4.77E-05
rs10052266	5	6010711	p15.32		G	A	0.12	0.32	0.094656	-2.01	4.83E-05
rs2763249	6	168926062	q27	SMOC2	A	G	0.22	-0.21	2.38E-01	1.97	4.89E-05

SNP-estimates are based on an additive model. Beta-estimates represent the increase or decrease FEV_1_/FVC in percentage.

Chr: Chromosome; All1: allele1; All2: allele2.

### Networks analysis

**Figure 1:**
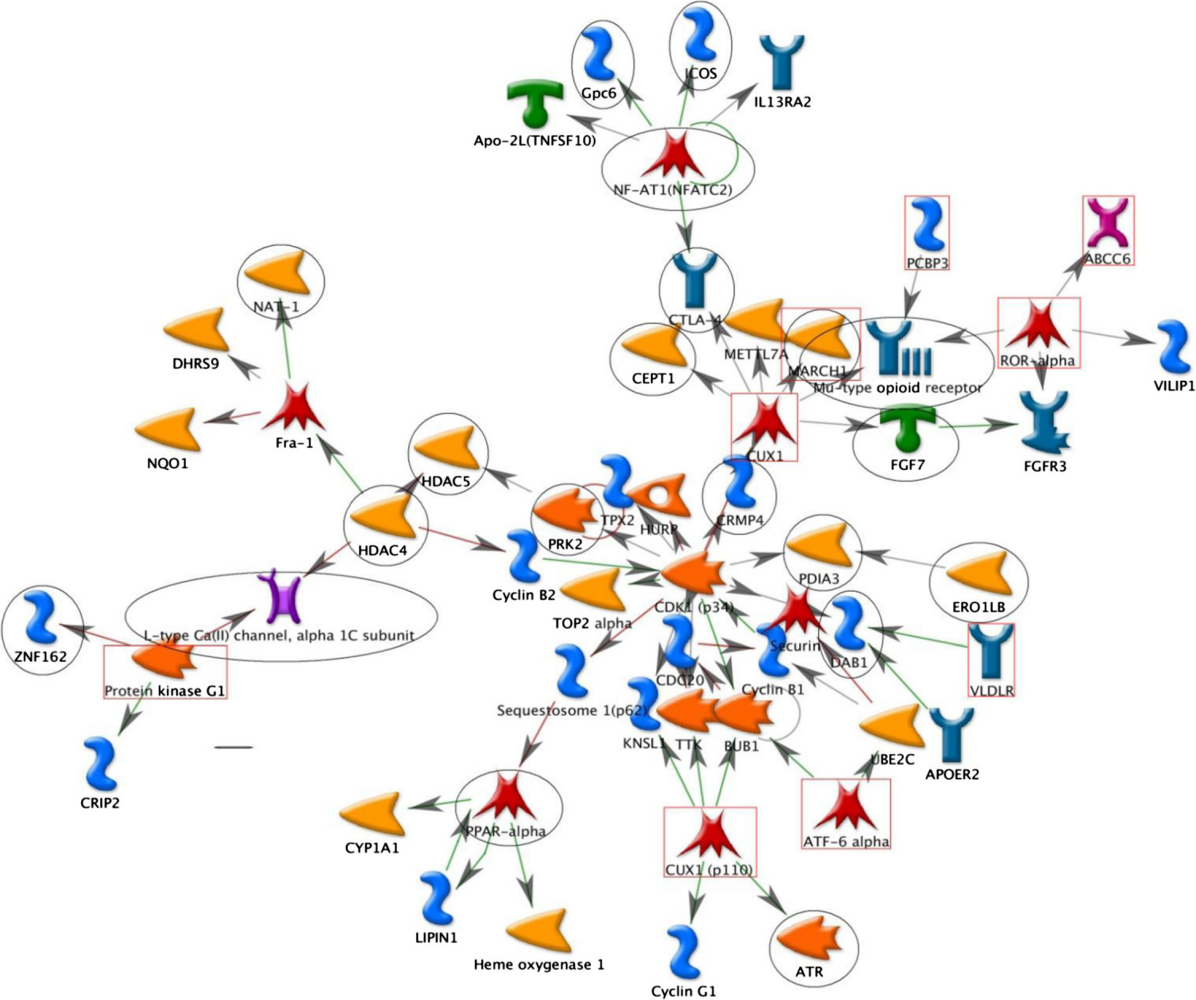
**Network for genes interacting with occupational exposure to affect lung function.** Network analysis revealed network clusters of genes with occupational exposure interaction effects on lung function (circled) that are connected by the network cluster built from genes affected by exposure to PM [27]. Genes modifying the occupational exposure effect on lung function are indicated by circles (FEV_1_) and rectangles (FEV_1_/FVC). All other symbols are explained in the Metacore legend (http://ftp.genego.com/files/MC_legend.pdf).

With a cut-off p-value of 10^-3^, we identified from our GWAS data 450 SNPs in 142 genes that interacted with occupational exposure to affect FEV_1_ and 360 SNPs in 116 genes that interacted with occupational exposure to affect FEV_1_/FVC. From our gene list, we found three network clusters consisting of 3, 6, and 13 genes. Integrating the gene list obtained from the gene expression study after exposure to PM, we found a network cluster consisting of 55 genes; the three clusters identified from our gene list are included in this big cluster (Figure 1). The genes obtained from the gene expression results linked all the network clusters together. Some key genes that interacted with occupational exposure in the network were *HDAC, CTLA-4*, *PPAR-alpha*, and *OPRM1*. These genes play important roles in connecting different network clusters into a big network cluster. In addition, we found that all network clusters were centered by the network cluster built from those genes identified from the expression study after exposure to PM.

## Discussion

To the best of our knowledge, this paper is the first study assessing gene by occupational exposure interactions on lung function using a community-based population. Our results from SNP-level analysis suggest that SNP rs9931086 in gene *SLC38A8* may significantly modify the effect of occupational exposure on FEV_1_. The results of network analysis suggest that genes *HDAC, PPAR-alpha,* and *CTLA-4* play important roles in the gene by occupational exposure interaction network.

The novel locus, SNP rs9931086, in gene *SLC38A8* had a significant gene by occupational exposure interaction effect on FEV_1_, although its genetic marginal effect was not statistically significant. For each C allele increase, the FEV_1_ decreased by 214 mL. We did not identify this gene through the network analysis; this may result, in part, from missing some genes that connect *SLC38A8* to the main gene network. For other top SNPs in our study, SNP rs6941466 in *SMOC2* (p = 3.21 × 10^-5^) was associated with FEV_1_/FVC through gene by occupational exposure interaction. *SMOC2* has been previously reported in both a gene main effect study [28] and a gene by smoking interaction study [23]. Another top SNP, rs1289714 (p = 5.25 × 10^-4^), in *HHIP* had a gene by occupational exposure effect modification on FEV_1_/FVC. The *HHIP* region was previously highlighted in the GWAS of FEV_1_/FVC focus on gene main effect using FHS data. The recent gene by smoking interaction on lung function study [23] discovered three gene regions, *DNER, HLA-DQB1/HLA-DQA2,* and *KCNJ2/SOX9*, with significant SNPs. However, the p-values for the SNPs in these genes were not statistically significant in our study.

The genes we identified through the network analysis are notable. Histone deacetylases (HDAC) are enzymes that regulate inflammatory gene expression. Several studies have shown the correlation between HDAC activity and COPD disease severity, measured by FEV_1_ and FEV_1_/FVC, and that HDAC activity is reduced in COPD patients [29-33]. Our results are consistent with a recent large-scale GWAS that identified an *HDAC4* association with lung function [34], which was also found in the gene by smoking interaction study [23].

Another inflammation-related gene, *PPAR-alpha*, is an isotype of the peroxisome proliferator-activated receptors, which can regulate the induction of inflammatory response [35]. One study showed that COPD patients had higher percentages of PPAR-alpha-positive alveolar macrophages and cells in their alveolar wall in COPD patients [36]. In addition, PPAR-alpha agonists may enhance the inducible isoform of nitric oxide synthase (iNOS) [37], and the expression of iNOS was found to increase in patients with severe COPD [38].

CTLA-4, the cytotoxic T-lymphocyte antigen 4, plays a role in downregulation of T-cell activation [39]. Further, the amount of T-cells correlates with the severity of airway obstruction and alveolar damage [40-43]. Genome-wide linkage analysis of the Boston Early-Onset COPD Study also showed a significant peak for airflow limitation on chromosome 2q, in the region of genes *CTLA-4* and *ICOS*[44,45]. *CTLA-4* gene polymorphisms are also associated with chronic bronchitis or COPD [46,47].

In the network analysis, the cluster network built by results of the gene expression experiment after PM exposure was associated with cell cycle regulation. This network of cell cycle regulation connected genes *HDAC, PPAR-alpha, CTLA-4* and may imply the possible underlying biological mechanism. Thus, the findings from SNP-level analyses and network analyses complement each other well. The genes found in our study are associated with inflammatory pathways. Genetic variation in the ability to mount an inflammatory response may explain varying individual responses to occupational exposure.

Importantly, we observed two genes through SNP-level analysis (*ZNF804A*) and network analysis (*OPRM1*) that interacted with occupational exposure to affect lung function. Although their functions remain to be uncovered, these two genes are significantly associated with many psychiatric diseases (e.g., schizophrenia, alcoholism, and tobacco use disorder) in several studies [48-53]. The comorbidity of abnormal pulmonary function or COPD and psychiatric diseases has been reported in many studies [54,55]. These psychiatric diseases might share some common pathway with COPD. However, we must exercise caution in interpreting these findings because smoking prevalence is higher among psychiatric patients [56]; nonetheless, another study controlling for smoking found schizophrenia to be an independent risk factor for COPD [57].

It should be noted that this study has several limitations. The first limitation is the lack of replication in population studies that use similar methods as the FHS, a community-based study with both occupational information and genome-wide genotyping. Therefore, our results are more exploratory in nature. Further, SNP-coverage was low for certain genes, which may result in missing several genes that might interact with occupational exposures. In addition, the mean FEV_1_ is higher in the group with highly likely dust exposure. This may be explained by gender distribution, since the proportion of males was higher in the group with high likelihood of dust exposure. Another potential explanation is the healthy worker effect confounding bias [58-60]. Participants in the highly likely dust exposure group were likely those in better health since most dusty jobs (e.g. heavy labor) require workers to be in better health. This bias may result in underestimation of the occupational exposure effect and reduce the effect size of our findings. Finally, our occupational exposure status was classified based on a JEM having less detailed occupational information than the UCSF COPD JEM; thus, our estimates may not reflect the true frequency. The occupational information in our study derives from a cross-sectional questionnaire, which may not reflect the longest-held job (e.g., participants may have worked in dusty job for 10 years but had switched to a non-dusty job by the time of the survey). These measurement errors might result in an underestimation of the gene by occupational exposure interaction association.

Our study indicates that integrating the results of a gene expression experiment into the GWAS of gene-environment interaction is informative for exploring novel genes. Future studies should increase the SNP coverage and measure occupational exposure more precisely by collecting more detail occupational information including the duration of each job and detail job titles. Moreover, future research could focus on examining the roles of *CTLA-4, HDAC*, and *PPAR-alpha* in the inflammatory airway process.

## Conclusions

Our study found that SNP rs9931086 in the *SLC38A8* gene and genes *CTLA-4, HDAC,* and *PPAR-alpha*, which are involved in inflammatory processes, may modify the effects of occupational exposure on lung function.

## Abbreviations

COPD: Chronic obstructive pulmonary disease; FEV1: Forced expiratory volume in one second; FHS: Framingham heart study; FVC: Forced vital capacity; GWAS: Genome-wide association study; JEM: Job exposure matrix; SNP: Single nucleotide polymorphism.

## Competing interests

The authors declare that they have no actual or potential competing financial interests.

## Author contributions

SL: Conception, data cleaning, analysis and interpretation of data, drafting and revising manuscript, final approval of manuscript; XL: Conception, analysis and interpretation of data, revision and final approval of manuscript; DCC: Conception, analysis and interpretation of data, revision and final approval of manuscript. All authors read and approved the final manuscript.

## Supplementary Material

Additional file 1: Figure S1 Manhattan plot for interacting SNP with occupational exposure on FEV1. **Figure S2**. Manhattan plot for interacting SNP with occupational exposure on FEV1/FVC. Click here for file
