# Supplementary material for: Gene-environment interaction effects on lung function- a genome-wide association study within the Framingham heart study
Source: Environ Health. 2013 Dec 1;12:101. doi: 10.1186/1476-069X-12-101 (PMC3882096; doi:10.1186/1476-069X-12-101)
Supplement: Additional file 1: Figure S1 — Manhattan plot for interacting SNP with occupational exposure on FEV1. Figure S2. Manhattan plot for interacting SNP with occupational exposure on FEV1/FVC. [file 1476-069X-12-101-S1.pdf]

Supplemental Material

Gene-environment Interactions on Lung Function- A Genome-Wide Association Study in the Framingham Heart Study

Shu-Yi Liao[1], Xihong Lin[1], David C. Christiani[1, 2]

[1] Harvard School of Public Health, Boston, MA

[2] Harvard Medical School, Boston, MA

FigureS 1. Manhattan plot for interacting SNP with occupational exposure on FEV_1

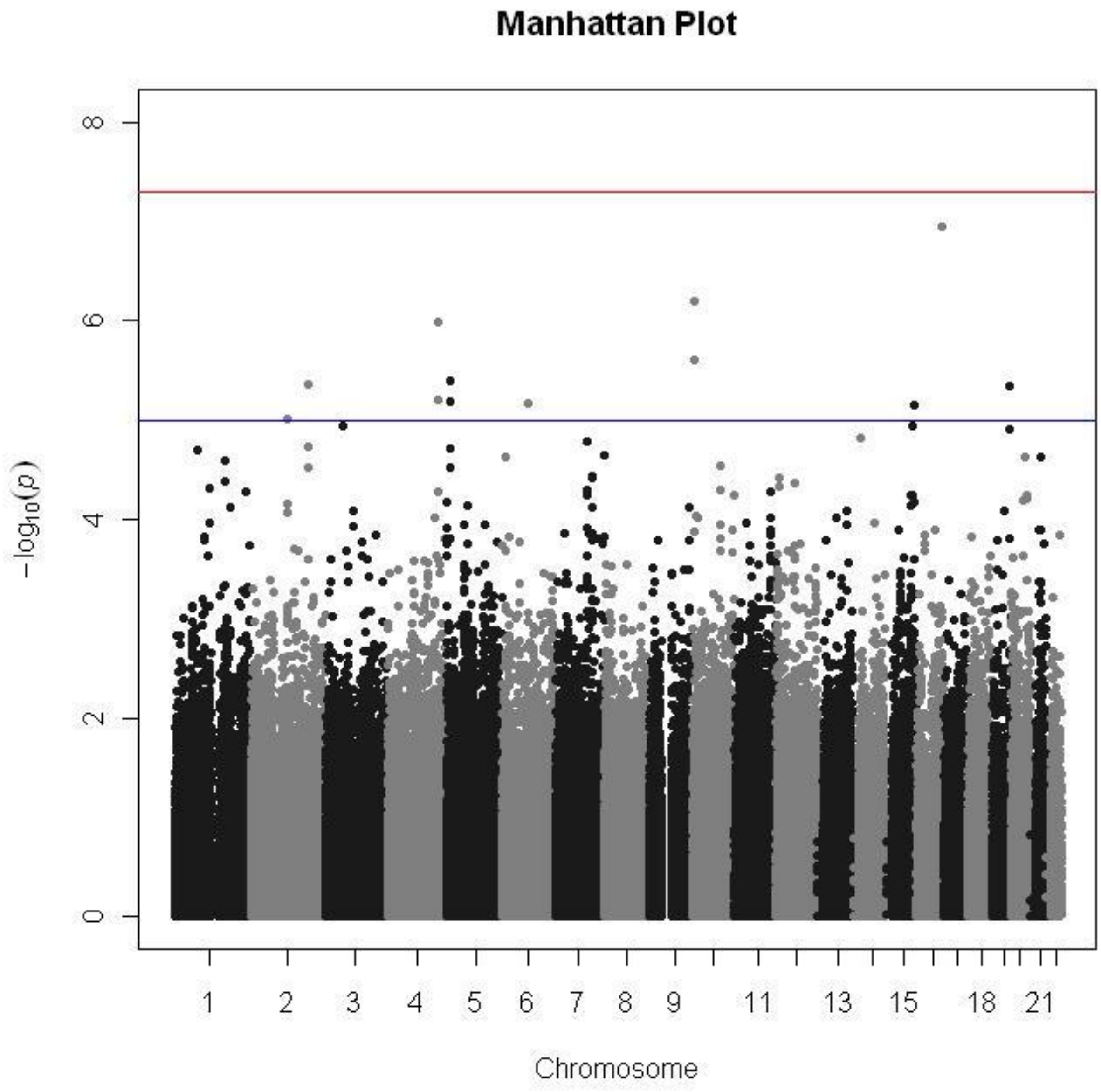
Manhattan Plot
$-\log_{10}(p)$
0
2
4
6
8
1
2
3
4
5
6
7
8
9
11
13
15
18
21
Chromosome

Figure S2. Manhattan plot for interacting SNP with occupational exposure on FEV_1/FVC

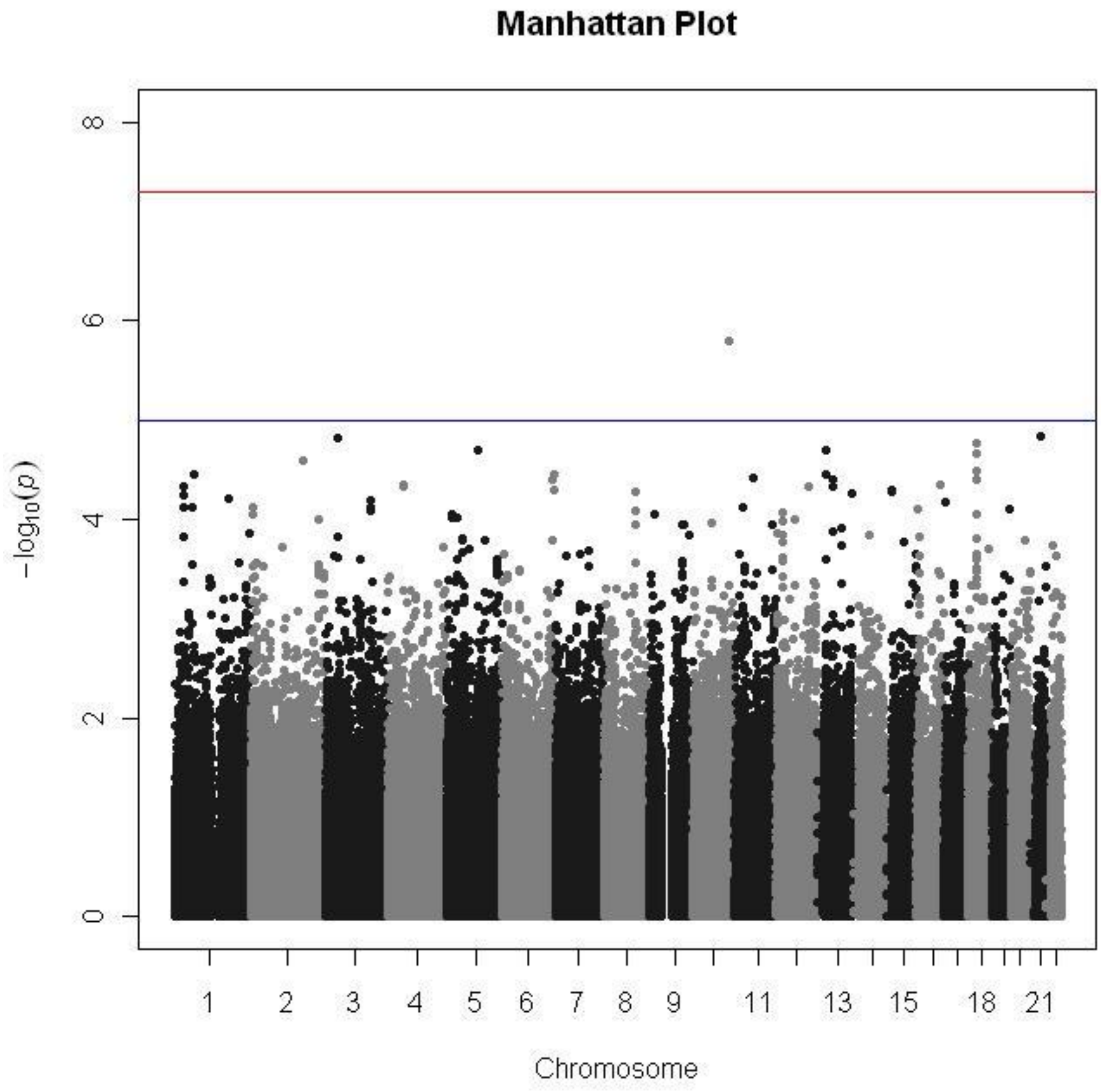
Manhattan Plot
$-\log_{10}(p)$
0
2
4
6
8
Chromosome
1
2
3
4
5
6
7
8
9
11
13
15
18
21
